# Belladonna as a Preventive of Scarlatina

**Published:** 1849-02

**Authors:** Samuel B. Robinson

**Affiliations:** Rutherford county, Tennessee


Art. VI.—*Belladonna as a Preventive of Scarlatina.* By Samuel B. Robinson, M.D., of Rutherford county, Tennessee.

I do not offer the following case as conclusive proof of the prophylactic powers of Belladonna in this disease, but as an instance favoring that doctrine as far as the trial of it in a single family goes. It appears to me worthy of publication, and I submit it in the hope that it will suggest similar experiments to other practitioners.

On the 8th of July last, I was called to see the little daughter of Capt. B., who was said to have scarlet fever, that disease having prevailed in this neighborhood all the forepart of the year. On my arrival I found the little patient presenting a pretty well-marked case of the disease, though not in its worst form. All the children in the family, seven or eight in number, had similar attacks. I learned from the parents that the little patient had just returned from a visit to her uncle, Major B.'s, where she was attacked. On the 13th of the same month, five days after my first visit to Captain B.'s, I was called to the house of the above named gentleman to see his little daughter, said to be laboring under scarlet fever. On my arrival I found the case to be one of decided malignity. The father being much alarmed at the prospect of the spread of the disease through his large family of children, I resolved, shortly after my arrival, to make an experiment with belladonna. Accordingly I dissolved three grains of the extract in an ounce of water, and gave to every child in the family three drops of the solution daily. Nearly six months have now elapsed and no case of scarlet fever, since the first, has occurred in Major R.'s family.

January 2, 1848.

